# Parameters of nonspecific resistance of calves with respiratory pathology before and after treatment

**DOI:** 10.5455/javar.2021.h522

**Published:** 2021-06-02

**Authors:** Hamdan Naef, Mohammad Abed Alhussen, Yury Anatolyevich Vatnikov, Liliya Valeryevna Cheskidova, Valentina Ivanovna Semenova, Pavel Andreevich Parshin, Mohammad Almohammad Alsalh

**Affiliations:** 1 Department of Veterinary Medicine, Peoples’ Friendship University of Russia (RUDN University), Moscow, Russia; 2 Federal State Budgetary Scientific Institution, Russian Ministry of Agriculture, All-Russian Veterinary Research Institute of Pathology, Pharmacology and Therapy, Farong, Russia; 3 Department of Animal Husbandry, College of Veterinary Medicine, Al Furat University, Deirez-Zor, Syria

**Keywords:** Respiratory diseases, natural resistance, treatment, calves

## Abstract

**Objective:**

The research was conducted to study the effect of a complex antimicrobial drug with an anti-inflammatory effect and an antimicrobial drug with an immunostimulating effect on the parameters of nonspecific resistance in calves.

**Materials and Methods:**

Two groups (*n* = 5 each) of sick calves with respiratory pathology were selected for this study. For the treatment of the first experimental group, a complex antimicrobial drug Sulfetrisan^®^ was used. The second experimental group of the calves was intramuscularly injected with the experimental drug gentaaminoseleferon (GIA). To assess the cellular component of immunity in the blood before and after treatment, the number of white blood cells, T-lymphocytes, B-lymphocytes, phagocytic activity of leukocytes, phagocytic number, and phagocytic index (PhI) were determined. In addition, for assessing the humoral component, serum complement activity (SCA), serum lysozyme activity, serum bactericidal activity (SBA), circulating immune complexes (CIC), and total immunoglobulins (total Ig) were measured. The results were compared with the baseline parameters of healthy calves of the control group.

**Results:**

When studying the parameters of the humoral and cellular components of nonspecific resistance, it was found that in sick animals, compared with healthy ones, respiratory pathology was accompanied by an imbalance in the immune system. In the process of recovery in animals of the experimental groups under the effect of the drugs, positive changes occurred. However, many of the studied parameters did not reach the values of healthy animals. In the group of calves that received GIA, compared with the calves given Sulfetrisan^®^, a significant increase in PhI (*p* < 0.05), SBA (*p* < 0.006), SCA (*p* < 0.05), total Ig (*p* < 0.0005), and CIC (*p* < 0.05) was observed, which indicated an increase in natural resistance due to the immunostimulating action.

**Conclusion:**

The use of GIA in sick animals added to an increase in the general nonspecific cellular and humoral resistance of calves, which made it possible to increase therapeutic efficacy and shorten their recovery time.

## Introduction

Bovine respiratory diseases (BRD) are widespread throughout the world and are ranked second among the pathologies of cattle in the Russian Federation [1,2]. At livestock enterprises, 30%–65% of young animals fall ill every year, and the death rate is 10%–31% of the cattle stock, which causes significant economic damage [3,4]. The occurrence, severity, and outcome of respiratory pathology depend on the etiology of the disease, the functional state of calves’ organisms, their housing and feeding conditions, and the adaptive ability of their organisms to the effects of adverse environmental factors [5,6].

The critical factors for respiratory diseases are infectious agents, which increase against various adverse factors. In addition, associations of microorganisms often cause respiratory diseases in calves [7,8]. Thus, viruses can induce immunosuppression, contributing to the development of secondary bacterial infections [9,10].

Complex adaptive mechanisms regulate the relationship among the organisms, microorganisms, environment, and the immune system [11,12]. Impaired function of the immune system is the main reason for the unfavorable development of the pathological process in BRD, which contributes to the occurrence of relapses, the transition to a chronic course, and an increase in complications [13,14]. Therefore, in clinical practice, to strengthen the immune system, it is necessary to use immunomodulatory drugs along with antimicrobial therapy [15,16].

Recently, antibacterial veterinary drugs combined with anti-inflammatory drugs, particularly glucocorticoids, have been widely used in the Russian Federation to treat BRD. This research has been conducted to study the effect of a complex antimicrobial drug, with an anti-inflammatory effect, and an antimicrobial drug, with an immunostimulating effect, on the parameters of nonspecific resistance of calves and to assess their effectiveness in the medication and treatment of respiratory pathology.

## Materials and Methods

### Ethical approval

The experiments on animals were approved by the Ethics Commission of FSBSI “All-Russian Veterinary Research Institute of Pathology, Pharmacology and Therapy” (FSBSI “ARVRIPP&T”) (No. 01-01-2019).

### Drugs for the treatment and cure of calves with respiratory pathology

On the farm, veterinarians used the complex drug Sulfetrisan^®^ in a solution for injections (“Api-San”, Moscow, Russia) to treat calves with BRD. As an active ingredient, it contains erythromycin (at 50 mg/ml) and a bacteriostatic antibiotic from the macrolide group, which inhibits RNA-dependent protein synthesis in bacteria. In addition, the antimicrobial effect of erythromycin was enhanced by the combination of Sulfadimethoxine (at 200 mg/ml) and Trimethoprim (at 18 mg/ml), which blocks two steps in the biosynthesis of nucleic acids and proteins necessary for bacteria (synthesis of dihydrofolic acid and dihydrofolate reductase activity). In the drug, the included combination of antimicrobial components gives a broad spectrum of antibacterial action against Gram-positive and Gram-negative microorganisms, and dexamethasone (at 0.1 mg/ml) has an anti-inflammatory effect [17,18].

According to the instructions, Sulfetrisan^®^ was prescribed to calves for bacterial etiology, which were responsive to the drug, intramuscularly at 5–10 ml per animal with an interval of 12–24 h severity of the disease. The complex injection drug “Gentaaminoseleferon” (GIA), which contains Gentamycin (40 mg/ml), the total antiviral activity of a combination of proteins of bovine recombinant Interferon alpha and Interferon-gamma (IFN-γ) (at least 1 × 10^4^ TCID_50_/ml), and aminoseleton (200 mg/ml) have been designed in FSBSI “ARVRIPP&T”.

Gentamycin is a widely used antibiotic from the aminoglycoside group [19,20]. Recombinant interferons obtained by the method of molecular biotechnology (“SPC ProBioTech”, Minsk, Republic of Belarus) are similar to interferons synthesized in the process of a protective reaction in response to antigenic effects in the organism of cattle. Aminoseleton is obtained by biotechnology from the spleen tissues of cattle, and it contains amino acids, macro- and microelements, which contribute to a rise in the general nonspecific resistance of the organism [21,22].

This drug is intended to cure infectious diseases in cattle of bacterial and mixed (bacterial–viral) etiology. For therapeutic purposes, GIA is intramuscularly administered at 1.0 ml per 10 kg of body weight every 24 h.

### Experimental design

The experiment was carried out on “Voronezhpish-cheprodukt” farm (Voronezh region) on Red-Motley calves aged 3–5-months weighing 75–88 kg. For the investigation, two groups of animals with clinical signs of respiratory pathology were formed.

According to the scheme adopted on the farm, the first animals of the experimental group (*n* = 5) were injected with Sulfetrisan^®^ intramuscularly: 10 ml per animal for 7 days with a 24-h interval. The second calves of the experimental group (*n* = 5) were intramuscularly injected with the experimental drug GIA at 1 ml per 10 kg of body weight for 7 days with a 24-h interval. A control group of healthy calves (*n* = 5) was used to determine the baseline parameters, which did not get drugs. For the research, blood was obtained from healthy calves before the experiment and from sick animals and 10 days after treatment. During the study, the calves were clinically observed to assess their general health status. The number of cured animals and the time of treatment and recovery were taken into account.

### Husbandry practices and ethics of experimental studies

The experiment was set up in September 2019. Before the investigation, the calves were kept in pens. After selecting sick animals into groups, they were transferred to a calf shed with the following microclimate parameters: temperature (12°C–18°C), relative air humidity (60%–75%), and air velocity (0.3–0.5 m/sec). Then, according to the standards with specialized feeds, the animals were fed, balanced in terms of the primary nutrients, and water was given *Ad libitum*.

### Diagnostics

The diagnosis was made comprehensively according to the results of clinical and laboratory studies. The general state of health (body temperature, food intake, behavior and habitus) was taken into account; the respiratory system was visually examined (respiratory rate, nasal discharge, type of breathing, shortness of breath, and dry/wet spontaneous cough), and auscultation (increased or decreased volume of breath sounds, rales, and signs of roaring).

Bacteriological and molecular identifications were carried out based on the research and development center of FSBSI “ARVRIPP&T”. Bacteriological examination of nasal lavages from sick animals was used to isolate *Staphylococcus* spp*.* and *Pasteurella multocida*, which were susceptible to gentamycin, erythromycin, and sulfadimethoxine. However, molecular identification did not detect the genomes of infectious bovine rhinotracheitis virus (IBRV), bovine viral diarrhea (BVDV), bovine parainfluenza virus type 3 (BPIV-3), and *Mycoplasma* spp*.*

Molecular genetic studies were carried out by PCR using approved test systems in accordance with the instructions. To identify the causative agent of IBRV, we used the RINOCOR test system; for BVDV, the VD test system was used, and for mycoplasmosis, the MIC-KOM test system (AmpliSens, Federal Budget Institution of Science “Central Research Institute of Epidemiology” of The Federal Service on Customers’ Rights Protection and Human Well-being Surveillance, Russia) was used. The test system for detecting BPIV-3 was VetBioChem, using a Rotor-Gene amplifier 6000 (Corbett Research, Mortlake, Australia).

Isolation and identification of microorganisms were carried out following the “Methodological guidelines for laboratory diagnosis of Pasteurellosis in animals and birds” and “Methodological guidelines for laboratory diagnosis of animal *Staphylococcosis*” approved in the Russian Federation. Smears were made, stained, and examined under a microscope at a magnification of 1,000× using a Bioscope-1 microscope (Russia) from the delivered material. *Staphylococcus* spp. were shown as large Gram-positive cocci, located in clusters in the form of bunches, and *Pasteurella* spp. were Gram-negative ovoids or short rods with rounded ends and noticeable bipolarity, around which a transparent capsule can be seen.

Material culturing was carried out in meat-peptone broth and meat-peptone agar (MPA). The morphological, cultural, biochemical, and pathogenic properties of the isolated cultures were studied. *Staphylococcus aureus* ferments glucose, Mannitol, maltose, and sucrose with the formation of acid without gas, without fermenting lactose and dulcite. However, it forms *β*-hemolysis on Neisler glucose–blood agar. All *Pasteurella* spp. are immobile, do not curdle milk, do not liquefy gelatin, reduce nitrates, and ferment (with the formation of acid without gas) glucose, mannose, and sucrose. On blood serum MPA or Hottinger’s Past agar, *P. multocida* does not cause hemolysis.

### Parameters of nonspecific resistance

ABX MICROS 60 hematology analyzer (Horiba, France) was used to determine the number of white blood cells (WBC). Immunological parameters, including serum complement activity (SCA), serum lysozyme activity (SLA), serum bactericidal activity (SBA), circulating immune complexes (CIC), and total immunoglobulins (total Ig), were determined using Shimadzu UV 1700 spectrophotometer (Shimadzu, Japan). Phagocytic activity of leukocytes (PhAL), phagocytic number (PhN), phagocytic index (PhI), number of T-lymphocytes, number of B-lymphocytes were measured using BIOSKOP-1 microscope (×1000; LOMO*,* St. Petersburg, Russia) [23].

### Statistical analysis

Mathematical processing of digital data was achieved using the descriptive statistics methods to the applied program Statistica 8.0 (Statsoft Inc., Tulsa, OK). The research results were expressed as mean (*M*) and standard error of the mean. Dissimilarity was considered statistically significant at *p* < 0.05, which was determined using the Wilcoxon paired W-test.

## Results and Discussion

When investigating the cellular component of nonspecific resistance (Table 1) in sick animals set against healthy animals, an increase in the number of T-cells (*p* < 0.0003) and B-cells (*p* < 0.00003) in the blood was found, which indicated the activation of the immune response in response to antigenic exposure. A decrease in PhAL (*p* < 0.002) demonstrated a reduction in the percentage of phagocytes able to bind to pathogenic microorganisms and participate in the elimination of CIC. A significant increase in WBC (*p* < 0.002) during inflammation is due to the need to provide the respiratory organs with phagocytes. Since the phagocytic activity of WBC is significantly reduced in comparison with healthy animals, the cellular defense begins to work in an extensive manner. This was partially compensated by an increase in the absorbing and digestive capacity of phagocytes, as evidenced by the rise in PhN (*p* < 0.002) and PhI (*p* < 0.02).

In the process of recovery in animals of the experimental groups, a significant decline in the number of WBC, T-cells, and B-cells was recorded. Even though these parameters did not reach the values of healthy animals, this trend indicated a decrease in the antigenic load on the cellular component of immunity under the effect of the antimicrobial components of Sulfetrisan^®^ and GIA drugs. However, a statistically significant increase in PhAL with a decrease in PhI and PhN, indicated the extensive functioning of the phagocytic system, which as a rule, led to a reduction in the organism’s resistance to diseases.

**Table 1.** Cellular factors of nonspecific resistance of calves.

Parameters	Healthy animals	Before treatment	Experimental group I	Experimental group II
WBC (10^9^/l)	9.5 ± 0.40	13.05 ± 0.72	9.8 ± 0.35**	10.7 ± 0.51*
PhAL (%)	82.82 ± 1.559	73.87 ± 1.300	86.25 ± 2.079**	85.95 ± 1.247**
PhI	5.90 ± 0.157	7.07 ± 0.189	5.43 ± 0.138**	5.82 ± 0.145**
PhN	4.90 ± 0.068	5.12 ± 0.054	5.00 ± 0.097	4.98 ± 0.135
T-**lymphocytes** (10^9^/l)	1.84 ± 0.069	2.45 ± 0.074	2.11 ± 0.035**	2.23 ± 0.113
B-lymphocytes (10^9^/l)	1.04 ± 0.033	1.50 ± 0.042	1.12 ± 0.032**	1.19 ± 0.057**

* Significant (*p* < 0.05).

** Significant (*p* < 0.005) in relation to the parameters before treatment.

**Table 2.** Humoral factors of nonspecific resistance of calves.

Parameters	Healthy animals	Before treatment	Experimental group I	Experimental group II
SBA (%)	86.83 ± 1.195	66.33 ± 1.256	70.83 ± 1.400*	77.83 ± 1.493**
SLA (μg/ml)	1.28 ± 0.038	1.37 ± 0.041	1.45 ± 0.027	1.53 ± 0.034*
SCA (%)	8.82 ± 0.187	7.15 ± 0.169	4.14 ± 0.227**	4.68 ± 0.170**
Total Ig (g/l)	27.70 ± 0.868	16.55 ± 0.804	21.28 ± 1.011**	27.72 ± 0.626**
CIC (g/l)	0.06 ± 0.004	0.08 ± 0.010	0.42 ± 0.021**	0.48 ± 0.023**

* Significant (*p* < 0.05).

** Significant (*p* < 0.005) in relation to the parameters before treatment.

At the same time, the first experimental group of calves had a significantly low PhI value (*p* < 0.05). In the second experimental group, the parameters of phagocytic activity (PhAL, PhN, and PhI) did not significantly differ from those of healthy animals, which proved the stimulating effect of GIA on phagocytosis.

When studying the humoral link of nonspecific resistance, it was found that in sick animals, compared with healthy ones, respiratory pathology was accompanied by an imbalance of the immune system (Table 2). Thus, a significant decrease in SBA was registered in serum (*p* < 0.00001), which on the one hand, might be due to a reduction in serum content of components that determined its bactericidal activity, and on the other hand, in a decrease of their antimicrobial action. An increase in the number of CIC and SLA was associated with an increase in antigens, and indicated an increase in the antibacterial activity of the antigen–antibody complex. However, a decline in SCA (*p* < 0.0002) and total Ig concentration (*p* < 0.00002) indicated that the organism’s resources were insufficient to respond to antigenic exposure and that the resistance of the calves’ organisms to infection was reduced.

In animals of both experimental groups, after treatment under the effect of the drugs, the same type of changes occurred. In the serum of calves after treatment with Sulfetrisan^®^ and GIA, compared with the sick animals, a high level of SBA, SLA, total Ig, and CIC was registered, which indicated the activation of humoral immunity. The decrease in SCA could be due to an increase in the consumption of components of the complement system for the formation of antigen–antibody–complement complexes (CIC).

Despite the positive dynamics of changes, the statistically low SBA was noted in the calves of both experimental groups compared to healthy animals. In addition, calves treated with Sulfetrisan^®^ also had lower total Ig levels (*p* < 0.001). However, in animals that received GIA, the total Ig level increased to baseline values, which confirmed the stimulating effect of some drug components on immunity.

Clinical assessment of the treatment of calves with respiratory pathology showed that when using Sulfetrisan^®^, 80% of the animals recovered during the experiment (four out of five), even though the isolated microflora was sensitive to the antimicrobial components. At the same time, the use of GIA provided 100% efficacy (all five calves recovered). There was no mortality in both groups, but after GIA therapy, the recovery time was reduced to 10.3 ± 0.42 days (12.7 ± 0.50 in case of treatment with Sulfetrisan^®^; *p* < 0.005).

It can be noted that in calves that received GIA, parameters of SBA (*p* < 0.006), SCA (*p* < 0.05), SLA, total Ig (*p* < 0.0005), CIC (*p* < 0.05), PhI (*p* < 0.05), number of WBC, and T-cells were higher, compared to the calves of the first experimental group. Since most researchers are inclined to believe that Gentamicin has immunosuppressive properties and suppresses the humoral immune response, the increase in the activity of natural resistance was due to the presence of interferon and aminoseleton in the drug [24]. Bovine recombinant interferons induce the endogenous cytokine system, activate cellular and humoral immunity [25]. In addition, the antibacterial role of IFN-γ, which is the activation of macrophages producing reactive oxygen and nitrogen species and prostaglandins that contributes to the development of an inflammatory process leading to the death of bacteria, has been proven [26,27]. Aminoseleton promotes a surge in the general nonspecific resistance of the organism and the intensification of metabolic processes has adaptogenic and antioxidant properties [21,28].

The use of Sulfetrisan^®^ in our experiment turned out to be insufficiently effective, apparently due to the presence of glucocorticoid in the drug, since it was believed that macrolide had an immunomodulatory effect and sulfonamides were capable of causing an excessive reaction of the immune system [29,30]. At the same time, dexamethasone, which simulates stressful stimuli, promotes a weakening of immune responses associated with impaired function of NK cells and macrophages, and a decrease in the level of cytokines and chemokines [31].

## Conclusion

The results of this research suggest that the use of antimicrobial agents in combination with glucocorticoids for the treatment of calves with respiratory pathology may not be effective enough due to the impaired functioning of their immune system. However, the use of the drug, which stimulates the general nonspecific resistance of sick animals, made it possible to increase therapeutic efficacy and reduce the recovery time. Thus, the use of immunostimulants in BRD positively affects animal health and will also reduce the dependence on antibacterial drugs.

## List of abbreviations

Gentaaminoseleferon (GIA), White blood cells (WBC), Phagocytic activity of leukocytes (PhAL), Phagocytic number (PhN), Phagocytic index (PhI), Serum complement activity (SCA), Serum lysozyme activity (SLA), serum bactericidal activity (SBA), Circulating immune complexes (CIC), Total immunoglobulins (total Ig), Interferon-gamma (IFN-γ), Bovine respiratory diseases (BRD), Infectious bovine rhinotracheitis virus (IBRV), Bovine viral diarrhea (BVDV), Meat-peptone agar (MPA), All-Russian Veterinary Research Institute of Pathology, Pharmacology and Therapy (FSBSI), Median Tissue Culture Infectious Dose (TCID), Polymerase Chain Reaction (PCR).
